# Nitric oxide system and diabetic nephropathy

**DOI:** 10.1186/1758-5996-6-17

**Published:** 2014-02-12

**Authors:** Bruno Schmidt Dellamea, Cristiane Bauermann Leitão, Rogério Friedman, Luis Henrique Canani

**Affiliations:** 1 Universidade Federal do Rio Grande do Sul, Porto Alegre, Brazil; 2 Endocrine Division of Hospital de Clínicas de Porto Alegre, Porto Alegre, Brazil

**Keywords:** Diabetes, Diabetic nephropathy, Polymorphism, eNOS, NOS-3, G894T, 4b/a, T786C

## Abstract

About 30% of patients with type 2 diabetes mellitus develop clinically overt nephropathy. Hyperglycemia is necessary, but not sufficient, to cause the renal damage that leads to kidney failure. Diabetic nephropathy (DN) is a multifactorial disorder that results from interaction between environmental and genetic factors. In the present article we will review the role of the nitric oxide synthase (NOS) in the pathogenesis of DN.

Nitric oxide (NO) is a short-lived gaseous lipophilic molecule produced in almost all tissues, and it has three distinct genes that encode three NOS isoforms: neuronal (nNOS), inducible (iNOS) and endothelial (eNOS).

The correct function of the endothelium depends on NO, participating in hemostasis control, vascular tone regulation, proliferation of vascular smooth muscle cells and blood pressure homeostasis, among other features. In the kidney, NO plays many different roles, including control of renal and glomerular hemodynamics. The net effect of NO in the kidney is to promote natriuresis and diuresis, along with renal adaptation to dietary salt intake.

The eNOS gene has been considered a potential candidate gene for DN susceptibility. Three polymorphisms have been extensively researched: G894T missense mutation (rs1799983), a 27-bp repeat in intron 4, and the T786C single nucleotide polymorphism (SNP) in the promoter (rs2070744). However, the potential link between eNOS gene variants and the induction and progression of DN yielded contradictory results in the literature.

In conclusion, NOS seems to be involve in the development and progression of DN. Despite the discrepant results of many studies, the eNOS gene is also a good candidate gene for DN.

## Introduction

About 30% of patients with type 2 diabetes mellitus develop clinically overt nephropathy [1]. Thus it appears that in humans hyperglycemia is necessary, but not sufficient, to cause the renal damage that leads to kidney failure. The risk is not linearly correlated to the duration of diabetes, with a decline after an initial progressive incidence, likely due to exhaustion of the subgroup of susceptible subjects [2,3].

Diabetic nephropathy (DN) is a multifactorial disorder that results from interaction between environmental and genetic factors. DN is histologically defined by thickening of the glomerular basement membrane, increased fractional mesangial volume, and podocyte abnormalities [2]. Hyperglycemia, hypertension and proteinuria are the main insults that cause structural abnormalities in a diabetic kidney [1,4-6].

The earliest known manifestation of diabetic kidney disease is the presence of small amounts of albumin in the urine, known as microalbuminuria. So far it is still unknown who will evolve to end-stage renal disease, and the genetic factor is a matter for debate and further study [7].

To better understand the pathogenesis of DN, nitric oxide (NO) must be considered, and the study of the polymorphism of genes involving it formation should be addressed.

### Nitric oxide system characterization

NO is a short-lived gaseous lipophilic molecule produced in almost all tissues and organs, a free radical exerting a variety of biological actions under both physiological and pathological conditions. NO is a paracrine mediator formed from its precursor L-arginine by a family of NO synthases (NOSs) with stoichiometric production of L-citruline. The NO system consists of three distinct NO synthase (NOS) isoforms, encoded by three distinct genes, including neuronal (nNOS or NOS-1), inducible (iNOS or NOS-2) and endothelial (eNOS or NOS-3). The gene encoding eNOS is located on chromosome 7 (7q35-q36) and contains 26 exons with an entire length of 21 kb [8-10].

### Nitric oxide system physiology

eNOS expression is regulated by transcription (changes in the rate of eNOS gene transcription), stabilization (alterations in eNOS mRNA stability), and phosphorylation [11]. The presence of these consensus sites is consistent with evidence showing that levels of eNOS transcripts are elevated by sheer stress, exercise and hypoxia [12]. Regulation of eNOS transcription by estrogens is still a matter of debate. Both lipopolysaccharide and tumor necrosis factor-α decrease eNOS gene expression by reducing the stability of eNOS-mRNAs. The constitutively expressed eNOS-mRNA is about 4052 nucleotides long and has a half-life of 10–35 h. Therefore, synthesis of the encoded proteins is likely to persist long after gene expression has been repressed [12]. Also, the activity of eNOS and the production of NO are diminished in senescent human endothelial cells [13].

The endothelium is a fundamental layer in the arterial wall both for the local regulation of flow to critical organs and for the protection of the vascular system from atherogenic insults. The correct function depends on the NO generation rate [14]. NO participates in regulatory functions including control of hemostasis, fibrinolysis, platelet and leukocyte interactions with the arterial wall, vascular tone regulation, vascular smooth muscle cell proliferation and blood pressure homeostasis. Disturbances in NO bioavailability have been found to cause endothelial dysfunction, leading to increased susceptibility to atherosclerotic lesion progression, hypertension, hypercholesterolemia, diabetes mellitus, thrombosis and stroke [15,16].

Insulin increases NO production, leading to vasodilatation and increased blood perfusion, and it also has anti-apoptotic and pro-survival effects on the ischemic/reperfused heart. Impairment of the phosphatidylinositide 3-kinases (PI3K) – protein kinase B (AKT) – eNOS – NO pathway as a manifestation of insulin resistance contributes to endothelial dysfunction, predisposing the endothelium to hyper-inflammatory and thrombotic states, while endothelin-1 expression and mitogenic effects are not affected [17]. Exercise, diet, cardiovascular drugs and insulin sensitizers, such as angiotensin-converting enzyme inhibitors, angiotensin receptor inhibitors, and the PPARg agonists, modulate both metabolic and cardiovascular effects of insulin simultaneously by regulating PI3K-AKT-eNOS signaling [18].

Data from animal models have suggested that eNOS null mice show a phenotype that resembles the human metabolic syndrome phenotype [19]. The oxidative effects of NO may play a role in insulin resistance and type 2 diabetes [20]. Two studies performed in a Spanish population found a positive association between e*NOS* polymorphisms and metabolic syndrome [21,22]. Another study, in an Italian population found an association with eNOS polymorphisms and insulin resistance [23]. Additionally, a positive association between G*894T* polymorphism and metabolic syndrome has been shown in Chinese and Japanese populations [24,25]. An association between G894TeNOS gene polymorphism and features of the metabolic syndrome was demonstrated in a southern Brazilian population, assuming a recessive model of inheritance [26].

### Nitric oxide system and kidney

NO must be considered in the pathogenesis of DN, since it plays numerous physiological roles in the kidney, including control of renal and glomerular hemodynamics, by interfering at multiple and physiologically critical steps of nephron function. NO dilates both the afferent and the efferent arteriole; it may augment the glomerular filtration rate (GFR) and influence renal sodium handling along various tubule segments from the thick ascending limb to the distal tubule and the collecting duct [27]. NO is also responsible for mediation of pressure natriuresis, maintenance of medullary perfusion, blunting of tubuloglomerular reabsorption, and modulation of renal sympathetic nerve activity [27,28]. The net effect of NO on the kidney is to promote natriuresis and diuresis, along with renal adaptation do dietary salt intake [29,30].

High levels of nNOs are expressed in macula densa and in minor intensity in specialized neurons within renal arteries of the hilus, arcuate and interlobular arteries. eNOs is strongly expressed in renal vascular endothelium, although tubular expression of eNOS also occurs. iNOS is weakly expressed in the kidney [27]. Changes in NOS gene expression do not always correlate well with measures of actual NO synthesis, because synthesis of NO by nNOS and eNOS is highly dependent on both adequate substrate and co-factor availability [31]. Changes in requirements for NO synthesis in the kidney often occur very fast, so regulation of total NOS expression seems not to play an important role, and it makes it more difficult to study molecular events in the regulation of NOS in the kidney [27,31,32].

The overall production of NO is decreased in chronic kidney disease (CKD), which contributes to cardiovascular events and further progression of kidney damage. There are many likely causes of NO deficiency in CKD, such as limitations on substrate (L-Arginine) availability, increased circulating levels of endogenous NOS inhibitors, in particular asymmetric dimethylarginine (ADMA). Reduced renal cortex abundance of the nNOS protein correlates with injury while increasing nNOS abundance may provide a compensatory, protective response [33].

In CKD, ongoing endothelial damage in the capillary system of the renal medulla and accompanying vascular rarefaction are thought to be central processes toward progressive kidney damage [34]. Reduced NO synthesis by endothelial cells due to accumulation of inhibitors of the eNOS, such as ADMA, has been pointed out as the cause of accelerating progression. Also, erythropoietin may have vasculoprotective effects on renal endothelium, which may be critically dependent on the activation of eNOS [35].

### Nitric oxide system and diabetic nephropathy

Recently, endothelial dysfunction has been commonly found in subjects with DN, and is considered the central pathophysiologic denominator for all cardiovascular complications of diabetes. In animal models of CKD and arteriosclerosis, blocking endothelial NO leads to an increase in microvascular disease, known to impair renal autoregulation [36].

Endothelial dysfunction has also been shown to lead to an uncoupling of the vascular endothelial growth factor (VEGF)-nitric oxide axis resulting in enhanced proinflammatory and proliferative effects of VEGF [36,37]. VEGF is increased in glomeruli and tubules in response to hyperglycemia. Whereas most studies have suggested that VEGF may be beneficial in non-diabetic renal disease, there is increasing evidence that VEGF may have a deleterious role in diabetic nephropathy. Endothelial dysfunction with NO deficiency may result in a loss of negative regulatory activity in the VEGF pathway. Consequently, VEGF may cause excessive endothelial cell proliferation, pathological macrophage infiltration, and overactivation of vascular smooth muscle cell, causing vascular injury [38,39].

Endothelin-1 is inhibited by vasodilators like NO and prostacyclins. Endothelin-1 acts in the kidney rising vascular resistance, which leads to reduction in blood flux, glomerular filtration rate and inhibition of salt and water reabsorption. It also causes glomerular cellular proliferation and accumulation of extracellular matrix [17].

NOS could be involved in the development of chronic diabetes complications through others pathways, such as uncoupling protein 2 (UCP2). UCP2 is expressed in several tissues, and protects against oxidative stress, in the regulation of insulin secretion by beta cells, and in fatty acid metabolism. Moreover, UCP2 preserves endothelial function through increasing NO bioavailability secondary to the inhibition of ROS production in the endothelium [40]. UCP2 has a potential role for inflammation and apoptosis regulation. These functions have major implications for cardiovascular and cerebrovascular chronic complications of diabetes. In fact, some UCP2 polymorphisms have been associated with the presence of diabetic chronic complications [41].

The metabolic abnormalities of diabetes cause mitochondrial superoxide overproduction in endothelial cells of both large and small vessels, enhancing five major pathways to diabetic complications: polyol pathway flux, activation of protein kinase C isoforms, overactivity of the hexosamine pathway, increased formation of advanced glycation end products and increased expression of the receptor for advanced glycation end products [38].

A broad spectrum of findings and issues has been amassed concerning the pathophysiology of the renal NO system in diabetes. Severe diabetes with profound insulinopenia can be viewed as a state of generalized NO deficiency. Available evidence suggests that diabetes triggers mechanisms that at the same time enhance and suppress NO bioavailability in the kidney [20,42]. It has been hypothesized that during the early phases of nephropathy, the balance between these two opposing forces is shifted toward increased NO [20,43,44]. This plays a role in the development of characteristic hemodynamic changes and may contribute to consequent structural alterations in glomeruli. The enhanced NO production may contribute to hyperfiltration and microalbuminuria that characterizes early diabetic nephropathy [42]. Both eNOS and nNO synthase can contribute to the altered NO production, particularly the first. As the duration of exposure to the diabetic milieu increases, factors that suppress NO bioavailability eventually prevail [20,44]. Increasing accumulations of advanced glycation end products may be one of the culprits in this process, leading to severe proteinuria, declining renal function, and hypertension [42]. In addition, this balance is continuously modified by actual metabolic control and the degree of insulinopenia [44].

Progression of early stages of DN to end-stage kidney disease is manifested by the gradual, inexorable scarring of the renal glomerulus followed by a similar fibrosing process in the tubulointerstitial region. Diabetic glomerular fibrosis is caused by accumulation of extracellular matrix proteins in the mesangial interstitial space resulting in fibrosis manifested by either diffuse or nodular changes [45]. The use of daidzein (caveolin inhibitor), hemin (hemoxygenase activator) or NO substrate in rats significantly decreases the renal cortical collagen content as compared to diabetic rats, presenting significant improvement in BUN, serum creatinine, proteinuria, urinary output, kidney weight/ body weight, renal cortical collagen content and nitrite/nitrate levels [46,47]. Although there is some controversy, most reports suggest higher levels of NO production early in diabetes but reduced levels in progressive DN [45,48].

The eNOS gene has been considerate as a potential candidate gene to DN susceptibility. Over the last few years, several polymorphisms of the eNOS gene have been identified, and their association with various diseases has been explored. Three polymorphisms have been extensively subject of research in efforts to identify genetic predisposition to chronic diabetes mellitus microvascular complications. These polymorphisms of interest in DN are the G missense mutation (rs1799983), a 27-bp repeat in intron 4, and the T786C single nucleotide polymorphism (SNP) in the promoter (rs2070744) [49-53]. However, not all studies support this association [54-56]. A recent meta-analysis [57], which analyzed these polymorphisms in the progression of DN, showed that G894T is significantly associated with DN, mainly in the allele contrast genetic model. However, in this meta-analysis patients with DN were compared to healthy subjects used as controls. Therefore, one cannot be sure if the polymorphisms were associated with DN or diabetes mellitus itself. In another meta-analysis [58], that compared diabetic patients without nephropathy (controls) to diabetic patients with nephropathy (cases), these polymorphisms were associated with increased risk for DN, supporting the involvement of the eNOS gene in the pathogenesis of DN.

The potential link between eNOS gene variants and the induction and progression of DN yielded contradictory results, exemplified by the association of the cited polymorphisms with ESRD and DN by some [49,51-54,59-69], but not by all studies [54-56,70]. G894T was linked to increased risk of macroalbuminuria and progression from microalbuminuria to macroalbuminuria, with declining glomerular filtration rate as serum creatinine value rises progressively, culminating in ESRD [66,67], independent of other risk factors.

These polymorphisms seem to change eNOS expression and to be associated with different levels of eNOS that make these associations clinically plausible. Intron 4 of eNOS contains a variable number of 27-pb consensus sequence repeats with the *b* allele having five repeats and the *a* allele having four repeats. A single nucleotide polymorphism affecting transcription of the eNOS promoter T786C reduces its activity to less than the half [71]. Plasma concentrations of NO metabolites are reduced in carriers of the “a” allele in intron 4 (intron with four repeats). The T786C SNP is strongly linked to the intron 4 polymorphism and functional studies reveal that the T786C mutation reduces eNOS gene promoter activity [50].

In conclusion, NOS seems to be involved in the development and progression of DN. Despite the discrepant results of many studies, the eNOS gene is also a good candidate gene for DN.

## Abbreviations

ADMA: Dimethylarginine; AKT: Protein kinase B; CKD: Chronic kidney disease; DN: Diabetic nephropathy; eNOS: Endothelial nitric oxide synthase; GFR: Glomerular filtration rate; iNOS: Inducible nitric oxide synthase; nNOS: Neuronal nitric oxide synthase; NO: Nitric oxide; NOS: Nitric oxide synthase; NOSs: Nitric oxide synthases; NOS-1: Neuronal nitric oxide; NOS-2: Induciblenitric oxide; NOS-3: Endothelialnitric oxide; PI3K: Phosphatidylinositide 3-kinases; UCP2: Uncouplingprotein 2; VEGF: Vascular endothelial growth factor.

## Competing interests

The author’s declare that they have no competing interests.

## Authors’ contributions

BSD reviewed the subject, searched for studies in databases, selected articles to include in the review and wrote the manuscript. CBL participated in the search and selection of articles and helped to draft the manuscript. RF helped in selection and helped to draft the manuscript. LHC conceived of the study, participated in coordination and helped to draft the manuscript. All authors read and approved the final manuscript.
